# Downregulation is the dominant effect of new regulatory mutations in a fungal pathogen

**DOI:** 10.1099/mgen.0.001769

**Published:** 2026-06-24

**Authors:** Ana Margarida Sampaio, Daniel Croll

**Affiliations:** 1 Laboratory of Evolutionary Genetics, Institute of Biology, University of Neuchâtel, 2000 Neuchâtel, Switzerland

**Keywords:** eQTL mapping, gene regulation, population genomics, *Zymoseptoria tritici*

## Abstract

Well-tuned gene regulation is essential for an organism’s survival. However, the ancestral state and predominant effects of regulatory mutations remain poorly understood outside of model fungi, such as Baker’s yeast with a highly compact genome organization. Here, we analysed a large panel of genome sequencing data of the major fungal wheat pathogen *Zymoseptoria tritici* to recapitulate the evolutionary history of *cis*-regulatory mutations. We found that new mutations are predominantly linked with the downregulation of the associated genes. The dominance of downregulation is reinforced for mutations occurring closest to the coding sequence and downstream. Mutations associated with the strongest downregulation segregate at high frequencies in populations despite their recent origin. This suggests that selection may have played a role in their rapid increase since speciation. Overall, our study highlights the power of mapping populations combined with genomic surveys to unravel fundamental patterns of regulatory evolution.

**Impact Statement**

How new mutations alter gene activity is a central question in biology, but most evidence comes from well-studied model organisms. Using a fungal pathogen responsible for major wheat crop losses worldwide, we traced the evolutionary history of regulatory mutations, i.e. changes in the genome that control gene expression. We found that new mutations predominantly switch genes down rather than up, particularly mutations near the gene itself. Surprisingly, the mutations associated with the strongest reductions in gene expression have spread widely across global populations, hinting at an adaptive advantage. This work broadens our understanding of how pathogens evolve and adapt through changes in gene regulation.

## Data availability

All sequence data are available from the NCBI Sequence Read Archive under the BioProject accession numbers PRJNA650267 (RNA-seq) and PRJNA250875-250876, 250889, 250915, 250917-250921, 250924-250925, 250927-250928, 250931-250932, 250935, 250937-250943, 250970, 250972, 250989-250995, 250997-251002, 251004-251007, 251049, 251056-251058, 251062, 251064, 251066, 299857, 327615, 331239-331255, 331257-331267, 331976-331991, 331993-332038, 335297-335306, 421484, 441050-441054, 442204-442207, 480739, 596434, 777581 and 870233 (DNA-seq).

## Introduction

 The regulation of gene expression is essential for the development and survival of organisms. Although organisms share conserved mechanisms of gene expression regulation, variation in gene expression profiles within and between species is common [1]. Genomic regions identified as expression quantitative trait loci (eQTLs) are important determinants of mRNA level variation within species [2,6]. eQTLs enable the association of DNA sequence variation with expression variation and, ultimately, the expression of complex traits. Most eQTLs act *in cis*, regulating the expression of neighbouring genes, typically resulting in larger gene expression effect sizes than *trans*-eQTLs, which affect distant loci across the genome [5,7]. The high prevalence of *cis*-eQTLs segregating within species and their typically strong effect highlight their importance in regulatory evolution over short timespans. However, the ancestral state and effects of mutations underpinning eQTLs within a species have largely remained unexplored.

Across the mutation spectrum, deletions and loss-of-function are generally more frequently observed in contrast to insertions or gene duplications with dosage effects [8,10]. This is explained, at least in part, by the likelihood of acquiring such mutations; however, tolerance of the organism to the mutational effects is also important. New regulatory mutations may also more likely disrupt transcriptional activation rather than causing a *de novo* gain in transcription, which leads to an overrepresentation of down-regulation effects [11,13]. While large-effect mutations on transcription may also, on average, be deleterious and selection should maintain such mutations at low frequencies by purifying selection [14,15], compensatory evolution may stabilize new *cis*-regulatory mutations by favouring *trans* mutations to reduce deleterious effects [16,17]. However, current insights in fungi remain largely limited to *Saccharomyces cerevisiae* with a highly compact genome and minimal intergenic space [18]. The compactness may constrain regulatory variation and, hence, the evolutionary trajectory and frequencies of large-effect mutations could differ in fungi with larger genomes.

Rapid regulatory evolution was shown to occur in filamentous fungal pathogens, such as the wheat pathogen *Zymoseptoria tritici*. To respond to strong environmental pressures, including the need to cope with host resistance and fungicide applications, plant pathogens have evolved a large body of regulatory mutations associated with gene expression variation. High genetic diversity and regulatory variation, in particular, is a hallmark of *Z. tritici* from the individual field scale to global diversity patterns [19,22]. A large body of *cis*-eQTLs were identified using a mapping population of field-collected isolates. Nearly two-thirds of all genes were found to segregate regulatory variants [23]. Most eQTLs were found within 2 kb up- and downstream of transcription starting sites (TSS), highlighting a dense network of regulatory mutations. The ancestral state of regulatory mutations at eQTLs remains unexplored, though.

Here, we characterized how newly arising *cis*-regulatory mutations impact gene expression at the population scale. Specifically, we assessed whether new mutations exhibit a predominant direction of expression change (down versus upregulation) and evaluated their genomic distribution across gene categories and distances to associated genes.

## Methods

### Expression QTL mapping population

A genome-wide map of regulatory polymorphisms governing gene expression has been established based on a collection of 146 *Z. tritici* isolates collected from a single field site in Switzerland [23]. Briefly, trimmed high-quality DNA short read sequences were aligned to *Z. tritici* reference genome IPO323 [24] using Bowtie2 (v2.3.4.3) [25] and used for variant calling based on the HaplotypeCaller integrated in GATK (v4.0.11.0) [26]. Called variants were filtered for quality with multiallelic sites being removed using bcftools (v1.9) [27]. Detailed filtering steps were reported previously [23]. The proportion of SNP and indel variants within 1 kb up- and downstream gene regions was obtained using bedtools (v2.27.1) [28]. RNA-seq datasets were trimmed and aligned to the *Z. tritici* reference genome IPO323 [24] using HISAT2 (v2.1.0) [29] with the parameter ‘–RNA-strandedness reverse’. RNA-seq datasets were here aligned to a second reference genome, Iran01_48b [30], to evaluate whether the identity of the reference could introduce read mapping and gene expression quantification biases. Normalization of the counts was performed using the –rpkm option implemented in the QTLtools run in –quan mode, identical to the procedure for IPO323 [23]. Pearson correlation coefficients of RPKM values per gene for each of the two reference genomes were performed per ortholog identified as reciprocal best blastn hits. By combining SNP calling data with expression data, *cis*-eQTLs were mapped using QTLtools (v 1.1) [31] using the *cis* conditional option and choosing a cis window of 10 kb equidistant from the TSS. All SNPs associated with mRNA abundance of a single, proximate gene were defined as *cis* eQTLs and used for further analyses, without filtering for association ranking (i.e. secondary eQTLs were retained if significantly associated). SNPs significantly associated with more than one gene were excluded. Characteristics of genomic locations of eQTLs were evaluated using bedtools intersect (v2.27.1) [28], intersecting SNP coordinates with IPO323 gene [32] and TE annotations [33]. Expression regression slope values produced by QTLtools were used to infer the direction of allelic effects on gene expression, with positive and negative values indicating increased and decreased expression relative to the reference allele, respectively.

### Global thousand-genome resequencing panel

We assessed global frequencies of regulatory mutations based on a large population sequencing panel covering all major wheat-producing regions where the pathogen is present. The thousand-genome panel comprises 1,035 genomes (Table S1, available in the online supplementary material) [19]. Variant calling was performed as described earlier [19] with SNPs mapped to the IPO323 reference genome using Bowtie2 (v2.4.1) [25]. We classified the associated *cis*-eQTL alleles as ancestral or derived using genome sequencing data of the closely related sister species *Zymoseptoria pseudotritici*. For this, we used publicly available PacBio long-read sequencing data from five *Z. pseudotritici* isolates from the species endemic region in Iran (NCBI SRA accessions SRR11972499-SRR11972503). Reads were aligned to the reference genome IPO323 [32] using minimap2 (v2.30) [34]. Variant calling was performed using bcftools (v1.22) [27]. Ancestral and derived allele states were assigned only at SNPs showing a consistent allele state across all five *Z. pseudotritici* isolates. The *Z. pseudotritici* allele was considered to be ancestral. *cis*-eQTL transcriptional effect sizes were obtained from QTLtools regression slope values.

## Results

**Fig. 1.**
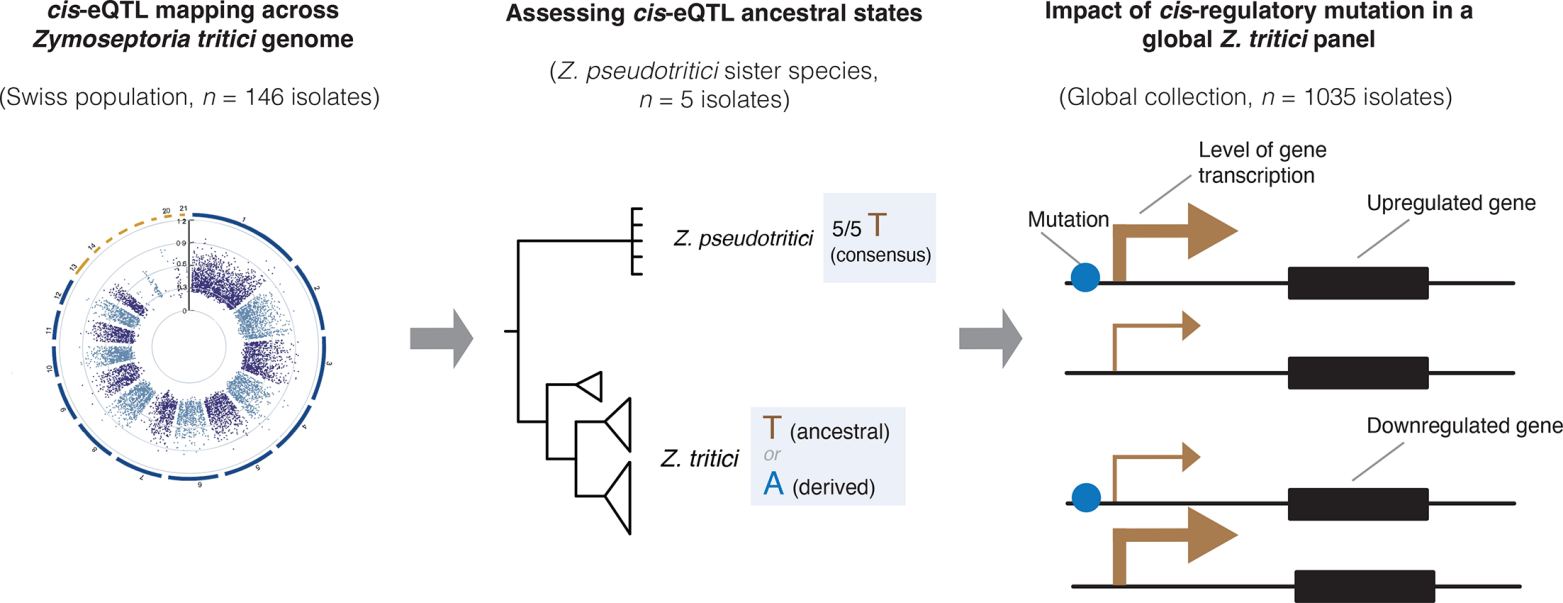
Schematic overview of the study design. *cis*-eQTLs have been mapped previously in a population of 146 *Z. tritici* isolates collected from an infected wheat field in Switzerland [23]. The circos plot identifies each significantly associated SNP locus with neighbouring gene expression (i.e. eQTLs). *cis*-eQTL ancestral states were assessed based on consensus genotypes in the sister species *Z. pseudotritici* (*n*=5 isolates). The impact of new mutations (i.e. derived alleles) in the associated genes was examined in a large genome panel of 1,035 *Z. tritici* isolates collected from multiple continents and covering the global distribution range of the pathogen.

We assessed the evolutionary history of regulatory mutations mapped in a field population of *Z. tritici*. Previously, using a mapping population established from 146 isolates recovered from an infected wheat field in Switzerland, *cis*-eQTLs were identified under standardized conditions for 65.3% of all genes [23] (Fig. 1). Here, we assessed the ancestral state at these *cis*-eQTL loci to distinguish the most likely recent mutation (i.e. derived allele) from the ancestral allele. We have performed this by searching for consensus genotypes at eQTL loci in the closest known sister species *Z. pseudotritici* (Table S2). We then used a global collection of a thousand *Z. tritici* genomes, covering all major regions where the pathogen has been identified [19], to quantify frequencies of ancestral versus derived *cis*-regulatory alleles (Fig. 1).

In the framework of the original eQTL mapping, RNA-seq reads were mapped to canonical reference genome produced for an isolate of European origin (IPO323) [23]. Here, we assessed whether the choice of the reference genome could introduce a reference bias in gene expression quantification. We remapped all RNA-seq reads to an alternative reference genome from an isolate collected close to the pathogen centre of origin (i.e. Iran). The independent mapping results revealed a very high correlation of RPKM values across all orthologous genes between the two genomes, indicating that, if present, any reference genome bias is minimal (Fig. S1 and Table S3). We also assessed a second potential source of bias caused by SNPs located in repetitive regions. We found that cis-eQTL loci were overwhelmingly located in gene-rich regions nearly devoid of transposable element sequences (Table S4). C*is*-eQTL mapping was restricted to SNPs, excluding indels (short insertion/deletion polymorphisms) as these were in high linkage disequilibrium [23] (Fig. 1). Most regulatory variants associated with *Z. tritici* gene expression were predominantly located close to transcription start sites [23]. SNPs accounted for 91.9% of all variants within 1 kb up- and downstream of genes. Previously, this indel depletion was suggested to reflect purifying selection against disruptive mutations in regulatory regions [19,23]. Among genes with a mapped *cis*-eQTL, most had a single *cis*-eQTL, with only 13% revealing two or more *cis-*eQTLs (Table S5).

**Fig. 2.**
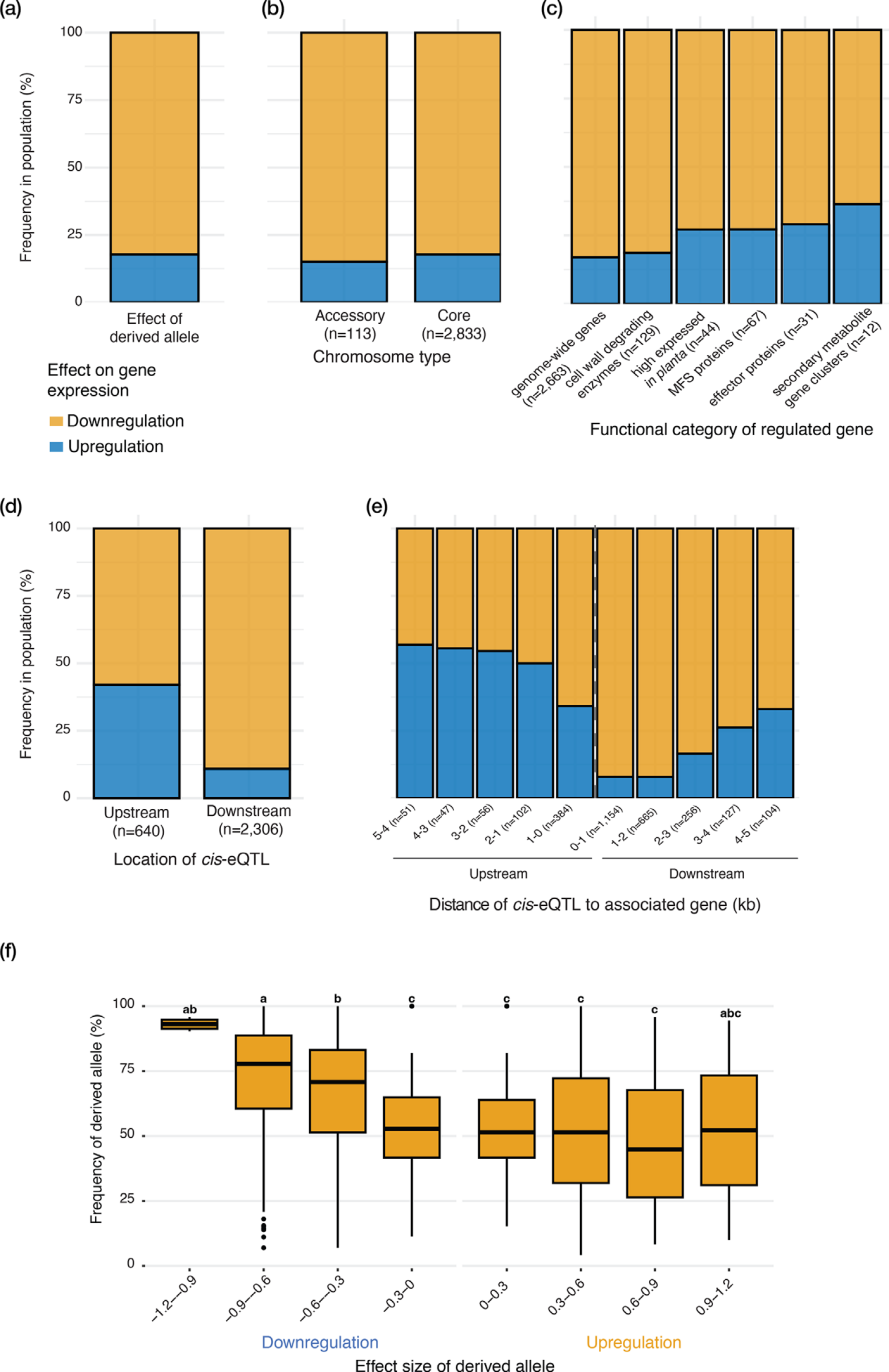
Frequency of down versus upregulation associations of new mutations at loci previously identified as *cis*-eQTL loci in the collection of genomes. (**a**) Overall effect of derived alleles on gene expression and separated by (**b**) chromosome type (core versus accessory), (**c**) regulated gene functions typically associated with pathogenesis in plants, including degradation of plant cell walls, toxin production encoded by metabolite gene clusters and effectors as previously described [23], (**d**) up- versus downstream position of the regulatory mutation, (**e**) specific locations of the regulatory mutation in 1 kb windows up- and downstream of the associated gene. *n* indicates the number of derived alleles per category. (**f**) *cis-*eQTL loci were binned based on the magnitude of the gene expression effect size (regression slope). Effect sizes were calculated as the association of derived alleles with gene expression (i.e. down- versus upregulation). For each effect size bin, the frequency of the derived allele is shown for the Swiss mapping population (*n*=146 isolates). Letters above each box denote Tukey's honest significance test groupings (α=0.05; 13 of 28 pairwise comparisons significant). Bins sharing a letter are not significantly different. One-way ANOVA: *F*(7, 2995)=54.46, *P*<2×10^⁻¹⁶^. For a data summary per allele frequency bins, see Fig. S3.

Derived alleles were more frequently associated with downregulation of the associated gene in the *cis*-eQTL mapping population. This pattern was even stronger in the global collection of genomes, where 82% of the new mutations (i.e. derived alleles) were associated with downregulation (Fig. 2a). To evaluate whether downregulation was a broad feature associated with new regulatory mutations, we examined expression associations across gene categories, as well as the distance between the eQTL and the target gene in the global collection of genomes (Table S5). Downregulation was similarly common for genes on core (82%) and accessory (85%) chromosomes (Fig. 2b). Similarly, downregulation was the most frequent effect of new mutations across gene categories previously shown to be likely associated with pathogenesis [23] (Fig. 2c). Interestingly, eQTLs for secondary metabolite gene clusters showed the strongest deviation from the genome-wide trend, with new mutations showing an ∼twofold higher enrichment for upregulation compared to the genome-wide average (Fig. 2c). New mutations located downstream of the TSS exhibited more frequent downregulation effects compared to mutations located upstream of the TSS (Fig. 2d). Furthermore, downregulation effects of new mutations were most dominant within 2 kb downstream of the TSS (Figs 2e and S2). Upregulation effects of new mutations increased both upstream and downstream of this interval. Hence, beyond this interval harbouring most *cis*-eQTLs [23], mutations in this interval also most frequently lead to downregulation.

Mutations with large effects on gene expression tend to occur at lower frequencies than expected under neutral evolutionary models [14,15]. Accordingly, we expected derived alleles causing strong downregulation to be rare. To address this question, we assessed how expression effects and allele frequencies correlate in the *cis*-eQTL mapping population (Table S6). New mutations associated with the strongest downregulation were showing among the highest allele frequencies in the mapping population (Figs 2f and S3). These results contrast with expectations that large-effect mutations should remain at low frequencies due to counterselection. We expanded these analyses to the global collection of isolates. For this, we selected *cis*-eQTL variants with the strongest downregulation effects (effect size <−0.8). We found a similar pattern to what was observed in the Swiss population, with *cis*-eQTLs exhibiting stronger regulatory effect sizes having a derived allele at high frequency in most clusters (Fig. S4). Interestingly, one of these *cis*-eQTL loci (1_4639299) has been previously reported as contributing to local adaptation [20]. The distribution of derived alleles at this locus suggests that the mutation was beneficial in North America and Oceania, which were colonized after the pathogen’s expansion from the Middle East to Europe [19].

## Discussion

Here, we characterized how newly arising *cis*-regulatory mutations impact gene expression in a fungal pathogen species. Our analyses show that new mutations are predominantly linked with downregulation of the associated genes. This effect is exacerbated if the regulatory mutation occurred downstream and in close proximity to the coding sequence. Interestingly, new mutations associated with strong downregulation are at high frequency in the population. Hence, such downregulation mutations might have been favoured by selection to reach high frequencies.

The observed bias towards downregulation is consistent with the fact that transcriptional disruption is more readily achieved by mutation than transcriptional enhancement [11,13]. Mutations with large effects are expected to be rare [14,15]. Consistent with this expectation *cis*-eQTLs of large effects tend to have low allele frequencies in the mapping population [23]. However, selection might act more strongly against expression increases, since they can impose higher metabolic cost or cause toxicity, reducing organism fitness [35,36], whereas moderate down-regulation may be more tolerated. Our analyses of ancestral states at eQTLs revealed that down-regulation was indeed the dominant effect of new mutations (i.e. derived alleles). Furthermore, these mutations rose to high frequency in the mapping population, as well as broadly across the pathogen’s global distribution range. Under stabilizing selection, large deviations from optimal expression, either up or down, should generally be deleterious and, therefore, rare. Hence, new mutations with strong effects should have remained at low frequencies rather than gaining such high frequencies. This suggests that the strong downregulation mutations may represent variants of weak or no fitness consequences. A small subset may have been favoured by selection if strong down-regulation was beneficial.

We analysed positional effects of *cis*-regulatory mutations associated with downregulation and found that these rose to higher population frequency if the eQTL was located downstream and close (< 2 kb) to the associated gene. These gene proximate regions are strongly enriched for cis-eQTLs [23]. More distal regulatory variants may more often be buffered by chromatin architecture or other epigenetic mechanisms [37,38]. The population frequency of down-regulation mutations was also associated with the function of the regulated genes. Genes encoding secondary metabolite clusters and candidate effectors involved in host interactions were more likely to have gained a mutation associated with upregulation compared to other categories, where downregulation is the dominant effect of new mutations. This observation may be consistent with several possible explanations. First, secondary gene clusters and pathogenicity-associated genes tend to be more likely controlled by epigenetic modifications rather than by changes in transcription factor binding, for example [39,40]. Such differences could have an impact on the likelihood of a new mutation to upregulate gene expression. Mutations could be associated, for example, with the insertion or excision of a transposable element with effects on chromatin organization [41]. Second, the emergence of *Z. tritici* as a major wheat pathogen from an ancestor with a possibly more endophytic lifestyle [42]. This transition could have selected for the upregulation of secondary gene clusters and other pathogenicity-related genes. Molecular genetics analyses will be required to disentangle the exact roles of regulatory mutations on genes and their causal role in the pathogen lifestyle.

The gain in frequency of new mutations associated with strong downregulation contrasts with findings that large-effect variants tend to be purged in the model yeast *S. cerevisiae* [43]. This discrepancy is likely explained by differences in genome architecture. *S. cerevisiae* has a highly compact genome, characterized by short intergenic distances and condensed promoter regions [18], which likely restricts regulatory variation to the immediate flanking regions of coding sequences [44]. In such a context, *cis*-regulatory mutations with strong effects are indeed more likely to disrupt core functions. Such deleterious mutations should then rapidly be removed by purifying selection. In contrast, the less compact genome of *Z. tritici* provides a broader *cis*-regulatory landscape with substantially enlarged intergenic distances. This architecture may enable mutational buffering, allowing more substantial expression changes without compromising core gene functions. Consequently, such mutations are more likely to be tolerated, enabling large-effect *cis*-variants to persist and reach higher frequencies across the pathogen’s global distribution range. Similar large-effect regulatory variation has been observed in rice (*Oryza sativa*) under environmental stress, where relaxed regulatory control enriched *cis*-acting mutations and expression variation [15]. While in rice such effects seem to be stress-dependent, in *Z. tritici* such expression variation seems constitutive, facilitating the retention and eventual fixation of large-effect mutations. The paucity of species with eQTL mapping information severely constrains comparative analyses of how genome architecture affects regulatory evolution.

In conclusion, we show that new *cis*-regulatory mutations predominantly result in gene downregulation and that such mutations have risen to high frequency. This highlights the power of combined population genomics and eQTL mapping approaches to investigate broad patterns of regulatory evolution.

## Supplementary material

10.1099/mgen.0.001769 Supplementary Material 1.

10.1099/mgen.0.001769 Supplementary Material 2.

## Abbreviations

eQTL: expression quantitative trait loci
TSS: transcription starting site

## References

[1] Zheng W, Gianoulis TA, Karczewski KJ, Zhao H, Snyder M (2011). Regulatory variation within and between species. Annu Rev Genomics Hum Genet.

[2] Boocock J, Alexander N, Tapia LA, Walter-McNeill L, Munugala C (2024). Single-cell eQTL mapping in yeast reveals a tradeoff between growth and reproduction. Elife.

[3] Mohanty JK, Jha UC, Dixit GP, Bharadwaj C, Parida SK (2024). eQTL-seq: a rapid genome-wide integrative genetical genomics strategy to dissect complex regulatory architecture of gene expression underlying quantitative trait variation in crop plants. *Plant Mol Biol Rep*.

[4] Osada N, Miyagi R, Takahashi A (2017). Cis- and trans-regulatory effects on gene expression in a natural population of *Drosophila melanogaster*. Genetics.

[5] Kita R, Venkataram S, Zhou Y, Fraser HB (2017). High-resolution mapping of *cis*-regulatory variation in budding yeast. Proc Natl Acad Sci U S A.

[6] Massouras A, Waszak SM, Albarca-Aguilera M, Hens K, Holcombe W (2012). Genomic variation and its impact on gene expression in *Drosophila melanogaster*. PLoS Genet.

[7] Lutz S, Brion C, Kliebhan M, Albert FW (2019). DNA variants affecting the expression of numerous genes in trans have diverse mechanisms of action and evolutionary histories. PLoS Genet.

[8] Katju V, Bergthorsson U (2019). Old trade, new tricks: insights into the spontaneous mutation process from the partnering of classical mutation accumulation experiments with high-throughput genomic approaches. Genome Biol Evol.

[9] Monroe JG, McKay JK, Weigel D, Flood PJ (2021). The population genomics of adaptive loss of function. Heredity (Edinb).

[10] Xu YC, Guo YL (2020). Less is more, natural loss-of-function mutation is a strategy for adaptation. *Plant Commun*.

[11] Hill MS, Vande Zande P, Wittkopp PJ (2021). Molecular and evolutionary processes generating variation in gene expression. Nat Rev Genet.

[12] Wittkopp PJ, Kalay G (2011). Cis-regulatory elements: molecular mechanisms and evolutionary processes underlying divergence. Nat Rev Genet.

[13] Wittkopp PJ, Haerum BK, Clark AG (2004). Evolutionary changes in cis and trans gene regulation. Nature.

[14] Josephs EB, Lee YW, Stinchcombe JR, Wright SI (2015). Association mapping reveals the role of purifying selection in the maintenance of genomic variation in gene expression. Proc Natl Acad Sci USA.

[15] Lye Z, Choi JY, Purugganan MD (2022). Deleterious Mutations and the Rare Allele Burden on Rice Gene Expression. Mol Biol Evol.

[16] Metzger BPH, Wittkopp PJ, Coolon JD (2017). Evolutionary dynamics of regulatory changes underlying gene expression divergence among saccharomyces species. Genome Biol Evol.

[17] Signor SA, Nuzhdin SV (2018). The evolution of gene expression in cis and trans. Trends Genet.

[18] Chen WH, Wei W, Lercher MJ (2011). Minimal regulatory spaces in yeast genomes. BMC Genom.

[19] Feurtey A, Lorrain C, McDonald MC, Milgate A, Solomon PS (2023). A thousand-genome panel retraces the global spread and adaptation of a major fungal crop pathogen. Nat Commun.

[20] Baril T, Puccetti G, Croll D (2025). Historic transposon mobilisation waves create distinct pools of adaptive variants in a major crop pathogen. Nat Commun.

[21] Sampaio AM, Tralamazza SM, Mohamadi F, De Oliveira Y, Enjalbert J (2025). Diversification, loss, and virulence gains of the major effector AvrStb6 during continental spread of the wheat pathogen *Zymoseptoria tritici*. PLoS Pathog.

[22] Singh NK, Karisto P, Croll D (2021). Population-level deep sequencing reveals the interplay of clonal and sexual reproduction in the fungal wheat pathogen *Zymoseptoria tritici*. Microb Genom.

[23] Abraham LN, Croll D (2023). Genome-wide expression QTL mapping reveals the highly dynamic regulatory landscape of a major wheat pathogen. BMC Biol.

[24] Goodwin SB, M’barek SB, Dhillon B, Wittenberg AHJ, Crane CF (2011). Finished genome of the fungal wheat pathogen *Mycosphaerella graminicola* reveals dispensome structure, chromosome plasticity, and stealth pathogenesis. PLoS Genet.

[25] Langmead B, Salzberg SL (2012). Fast gapped-read alignment with Bowtie 2. Nat Methods.

[26] Van der Auwera G, O’Connor B, Safari (2020). Genomics in the cloud: using docker, GATK, and WDL in terra. O’Reilly Media.

[27] Danecek P, Bonfield JK, Liddle J, Marshall J, Ohan V (2021). Twelve years of SAMtools and BCFtools. Gigascience.

[28] Quinlan AR, Hall IM (2010). BEDTools: a flexible suite of utilities for comparing genomic features. Bioinformatics.

[29] Zhang Y, Park C, Bennett C, Thornton M, Kim D (2021). Rapid and accurate alignment of nucleotide conversion sequencing reads with HISAT-3N. Genome Res.

[30] Badet T, Oggenfuss U, Abraham L, McDonald BA, Croll D (2020). A 19-isolate reference-quality global pangenome for the fungal wheat pathogen *Zymoseptoria tritici*. BMC Biol.

[31] Delaneau O, Ongen H, Brown AA, Fort A, Panousis NI (2017). A complete tool set for molecular QTL discovery and analysis. Nat Commun.

[32] Lapalu N, Lamothe L, Petit Y, Genissel A, Delude C (2025). Improved gene annotation of the fungal wheat pathogen *Zymoseptoria tritici* based on combined iso-seq and RNA-Seq evidence. Mol Plant Microbe Interact.

[33] Baril T, Croll D (2023). A pangenome-guided manually curated library of transposable elements for *Zymoseptoria tritici*. BMC Res Notes.

[34] Li H (2018). Minimap2: pairwise alignment for nucleotide sequences. Bioinformatics.

[35] Tomala K, Korona R (2013). Evaluating the fitness cost of protein expression in Saccharomyces cerevisiae. Genome Biol Evol.

[36] Kemble H, Eisenhauer C, Couce A, Chapron A, Magnan M (2020). Flux, toxicity, and expression costs generate complex genetic interactions in a metabolic pathway. Sci Adv.

[37] Stadhouders R, van den Heuvel A, Kolovos P, Jorna R, Leslie K (2012). Transcription regulation by distal enhancers: who’s in the loop?. Transcription.

[38] Floc’hlay S, Wong ES, Zhao B, Viales RR, Thomas-Chollier M (2021). *Cis*-acting variation is common across regulatory layers but is often buffered during embryonic development. Genome Res.

[39] Nanchira Abraham L, Sampaio AM, Bhattacharyya S, Moser Tralamazza S, Croll D Intra-population variability in genome-wide repressive histone marks underpins differential gene expression in a fungal wheat pathogen. Genomics.

[40] Soyer JL, El Ghalid M, Glaser N, Ollivier B, Linglin J (2014). Epigenetic control of effector gene expression in the plant pathogenic fungus *Leptosphaeria maculans*. PLoS Genet.

[41] Meile L, Peter J, Puccetti G, Alassimone J, McDonald BA (2020). Chromatin dynamics contribute to the spatiotemporal expression pattern of virulence genes in a fungal plant pathogen. mBio.

[42] Sánchez-Vallet A, McDonald MC, Solomon PS, McDonald BA (2015). Is *Zymoseptoria tritici* a hemibiotroph?. Fungal Genet Biol.

[43] Ronald J, Akey JM (2007). The evolution of gene expression QTL in *Saccharomyces cerevisiae*. PLoS One.

[44] Spivak AT, Stormo GD (2016). Combinatorial cis-regulation in saccharomyces species. G3: Genes Genom Genet.

