# Supplementary material for: Downregulation is the dominant effect of new regulatory mutations in a fungal pathogen
Source: Microb Genom. 2026 Jun 24;12(6):001769. doi: 10.1099/mgen.0.001769 (PMC13293413; doi:10.1099/mgen.0.001769)
Supplement: Supplementary Material 1. [file mgen-12-01769-s001.pdf]

Supplementary Figures

Sampaio et al.

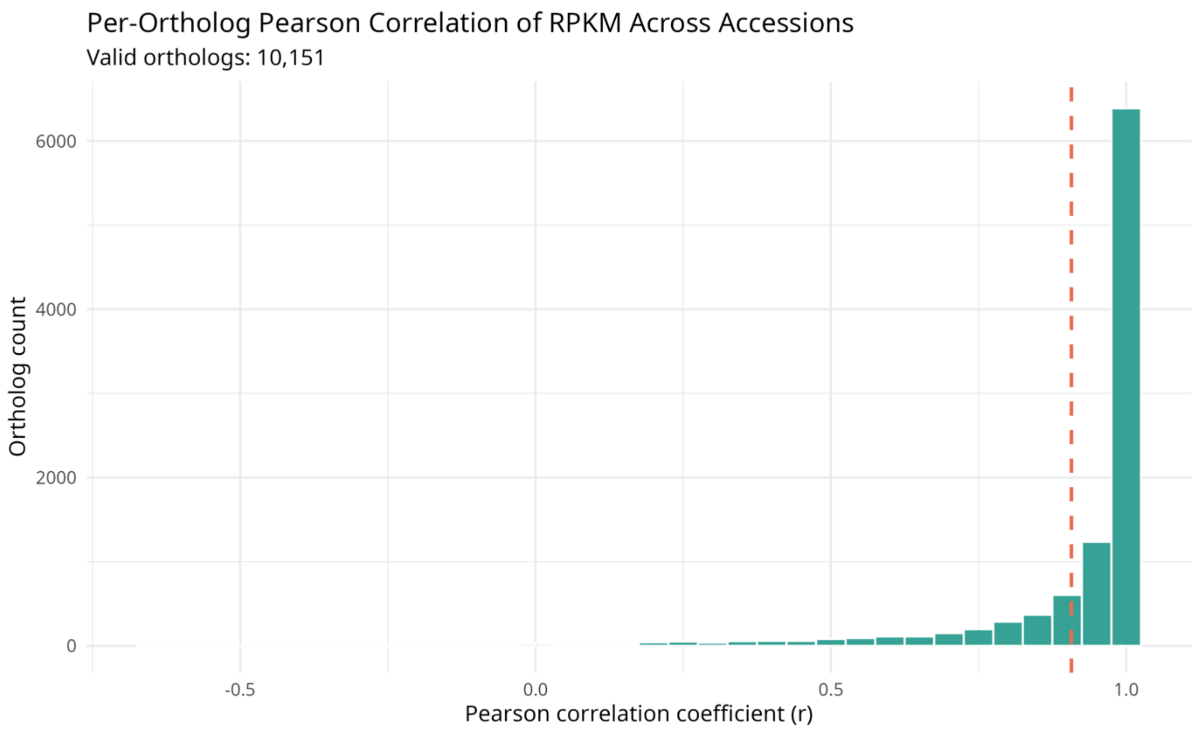

Supplementary Figure S1: Distribution of per-ortholog gene Pearson correlation of RPKM values between *Z. tritici* IPO323 and Iran01_48b reference genomes.

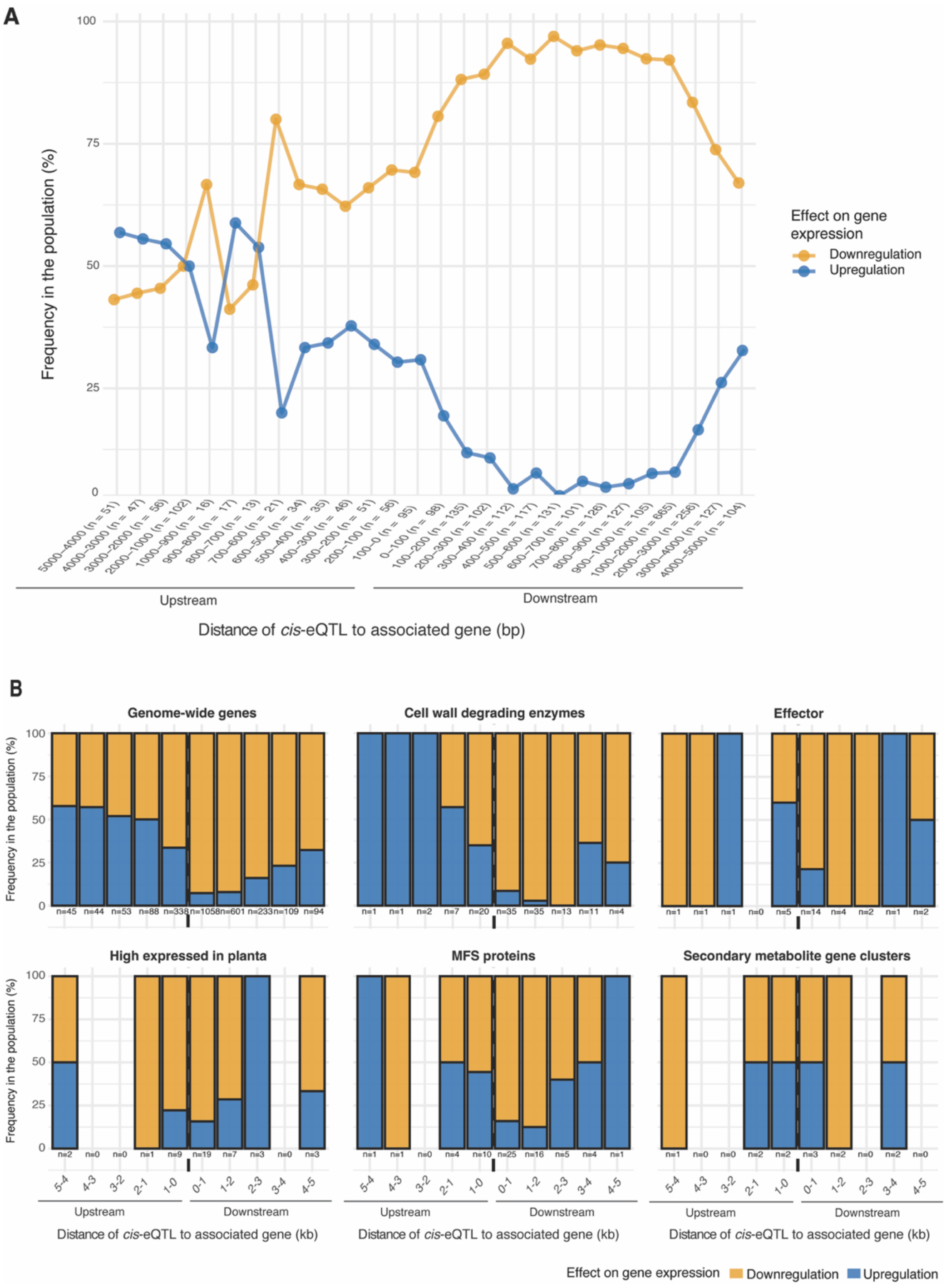

Supplementary Figure S2: Frequency of down *vs.* upregulation effects of new mutations at loci previously identified as *cis*-eQTL loci in the global genome panel by: A) *cis*-eQTL distance to associated gene. B) *cis*-eQTL distance to associated gene, per regulated gene functions.

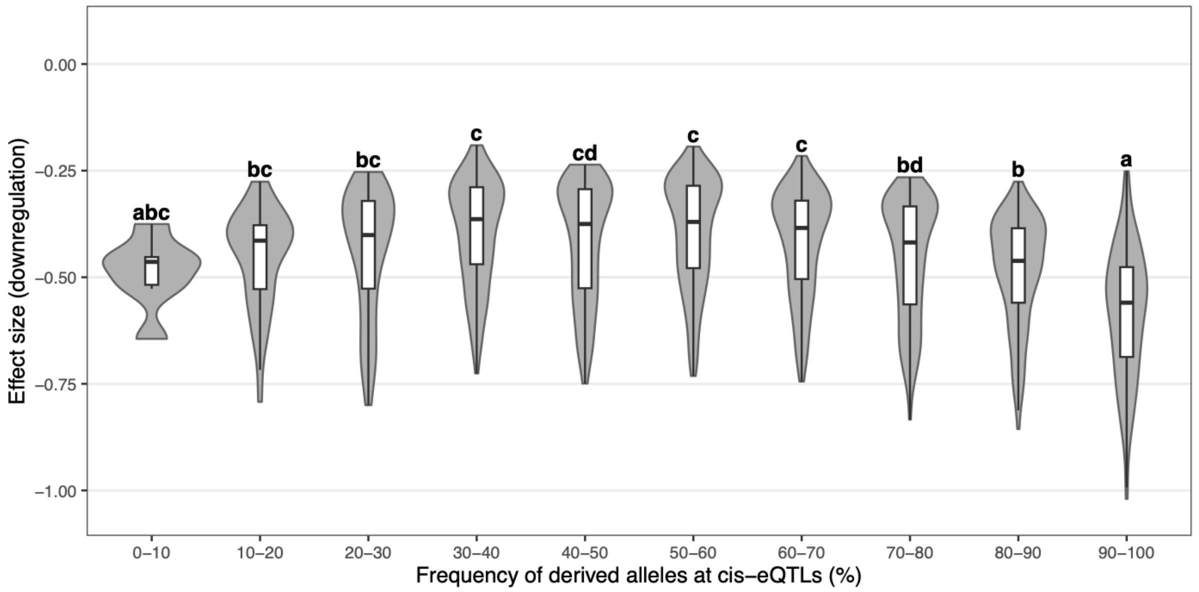

Supplementary Figure S3: Violin plots showing the distribution of *cis*-eQTL effect sizes for the derived allele across bins of derived allele frequency (10% intervals) in the Swiss population (*i.e.* eQTL mapping population). Negative effect sizes (assessed by regression slopes) indicate that the derived allele was associated with reduced expression. Boxes indicate interquartile range and median. Letters above each violin denote Tukey HSD groupings ($\alpha = 0.05$; 14 of 45 pairwise comparisons significant). Bins sharing a letter are not significantly different. One-way ANOVA: $F\ (9,\ 1656) = 29$, $p < 2\times10^{-16}$.

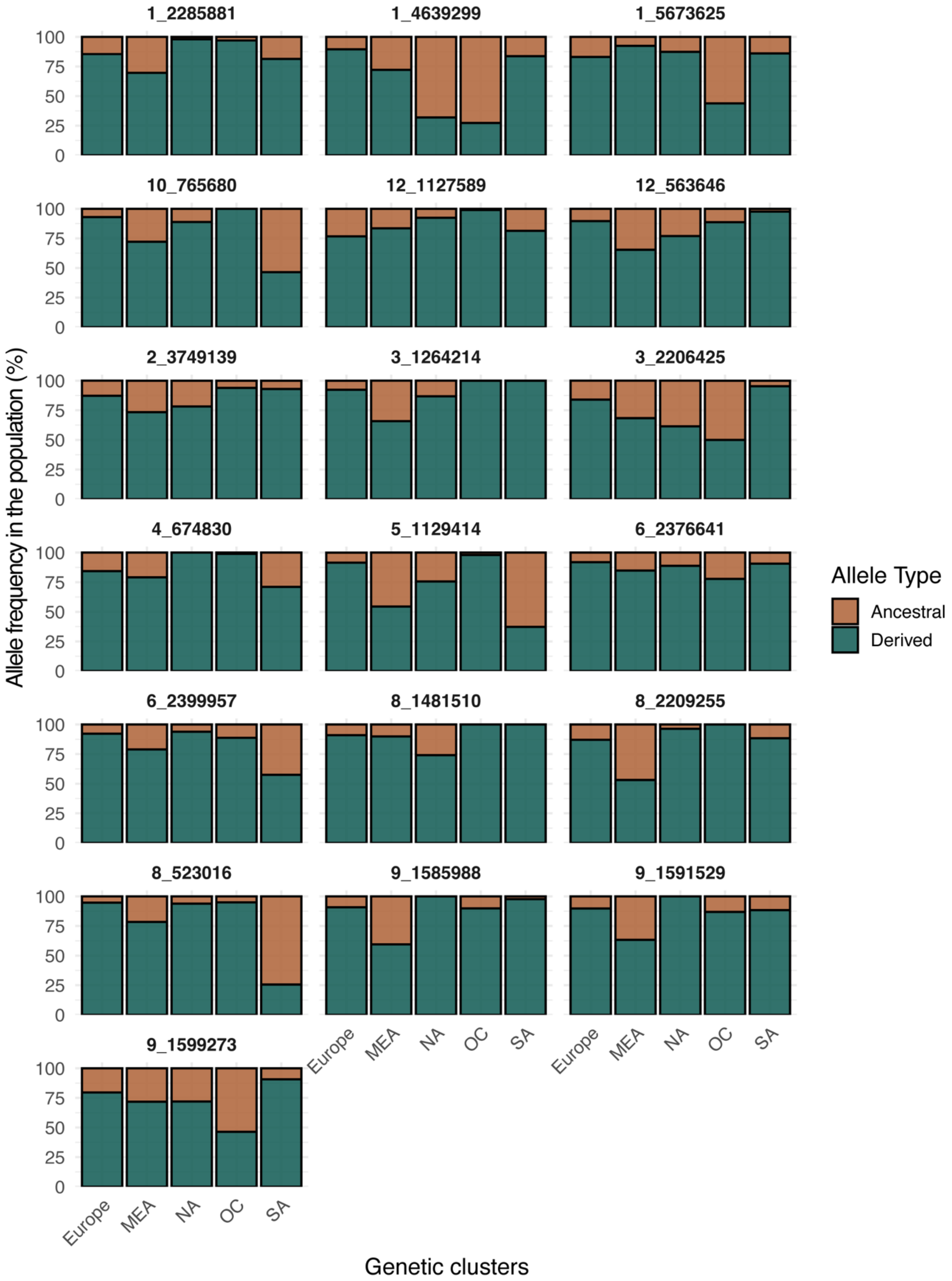

Supplementary Figure S4: Frequency of ancestral and derived alleles at *cis*-eQTLs with strongest down-regulation effect sizes (effect size < -0.8). Numbers indicate chromosome and respective position of the alleles. Genetic clusters: MEA – Middle East, NA – North America, OC – Oceania, SA – South America.
